# Arthroscopic Technique for Autologous Minced Cartilage Implantation for Focal Lesions of the Capitellum Humeri of the Elbow: A Technical Note

**DOI:** 10.1002/atn2.70230

**Published:** 2026-08-13

**Authors:** Tim Leschinger, Nadine Ott, Sebastian Wegmann, Maximilian Weber, Lars P. Müller, Tamara Babasiz

**Affiliations:** ^1^ Department of Orthopaedic and Trauma Surgery Faculty of Medicine University Hospital Cologne, University of Cologne Cologne Germany

## Abstract

Focal cartilage defects of the capitellum humeri are frequently underestimated, despite their substantial contribution to elbow pain and functional impairment in young and physically active patients. Arthroscopic autologous minced cartilage implantation is a single‐stage biological cartilage repair technique that has shown favorable clinical and imaging outcomes in larger joints. Osteochondral lesions of the capitellum humeri of the elbow, particularly in the setting of osteochondritis dissecans, represent a challenging condition in young and physically active patients. However, detailed descriptions of the arthroscopic surgical technique and its application in the elbow joint remain limited. Therefore, the purpose of this Technical Note is to describe a step‐by‐step arthroscopic technique for autologous minced cartilage implantation in focal osteochondral lesions of the capitellum humeri. Indications, surgical pearls and pitfalls, and postoperative care are outlined, and the potential advantages of this technique in the treatment of elbow cartilage lesions are discussed.

## Graphical abstract

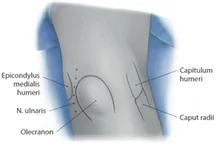

**VIDEO 1:** Arthroscopic autologous minced cartilage implantation for a focal osteochondral lesion of the capitellum humeri is presented. The procedure is shown in the right elbow, with the patient positioned in the lateral decubitus position and the operative arm supported in an arm holder. Important anatomical landmarks are marked before portal placement. Arthroscopy is initiated through a high posterolateral viewing portal, followed by establishment of a low posterolateral working portal and an anterolateral portal to access the radiocapitellar joint. Diagnostic arthroscopy is performed, hypertrophic posterolateral plica tissue is resected when present, and the capitellar osteochondral lesion is probed to assess fragment stability. Unstable cartilage and osteochondral fragments are mobilized and removed arthroscopically, with cartilage tissue harvested using a shaver while preserving the subchondral bone. The defect bed is debrided to stable vertical cartilage margins, and the lesion is carefully dried. The harvested cartilage is mechanically minced, combined with autologous platelet‐rich plasma (PRP), and delivered into the defect through an application cannula. The graft is molded with a blunt elevator to achieve uniform filling without overfilling. Thrombin is then applied to induce fibrin formation and stabilize the construct, followed by final sealing with an additional fibrin layer. Gentle passive elbow range‐of‐motion testing confirms construct stability. Video content can be viewed at https://doi.org/10.1002/atn2.70230.

Single‐stage biological cartilage repair techniques have gained increasing attention as alternatives to graft‐based or cell‐expanded procedures. Autologous minced cartilage implantation allows intraoperative harvest and reimplantation of cartilage tissue without the need for additional donor sites or staged surgery.^1^ Although this technique has been described in detail for other joints, such as the knee, ankle, or shoulder,^2^, ^3^, ^4^ technical descriptions tailored to the anatomical and biomechanical characteristics of the elbow joint is missing.

Osteochondral lesions of the capitellum humeri represent a frequent indication for arthroscopic intervention in young and physically active patients. In advanced or unstable cases, surgical treatment is required to address pain, mechanical symptoms, and functional impairment while preserving joint congruency and range of motion.^5^, ^6^ A variety of surgical options have been described, including fragment fixation, debridement with bone marrow stimulation, and osteochondral grafting procedures. However, each of these techniques is associated with specific limitations, such as donor‐site morbidity, technical complexity, or restrictions during postoperative rehabilitation.^7^

Autologous minced cartilage implantation is indicated for focal osteochondral lesions of the capitellum humeri or radial head with intact surrounding cartilage margins, including in situ or detached fragments and intra‐articular loose bodies. Contraindications include diffuse or bipolar cartilage degeneration, corresponding cartilage lesions on the opposing joint surface, bilateral osteochondral defects, inflammatory joint disease, and insufficient autologous cartilage tissue for implantation.^8^, ^9^

Arthroscopic application of minced cartilage in the elbow requires precise patient positioning, portal placement, controlled defect preparation, and careful handling of the cartilage fragments to achieve stable defect filling while avoiding damage to the surrounding cartilage and subchondral bone. Given the confined joint space and the proximity of neurovascular structures, a standardized and reproducible surgical approach is essential. Step‐by‐step instructions are presented for the arthroscopic technique for autologous minced cartilage implantation for focal osteochondral lesions of the capitellum humeri, highlighting key technical aspects, pearls, and potential pitfalls specific to the elbow joint.

## SURGICAL TECHNIQUE

### Preoperative Planning and Patient Positioning

**FIGURE 1:**
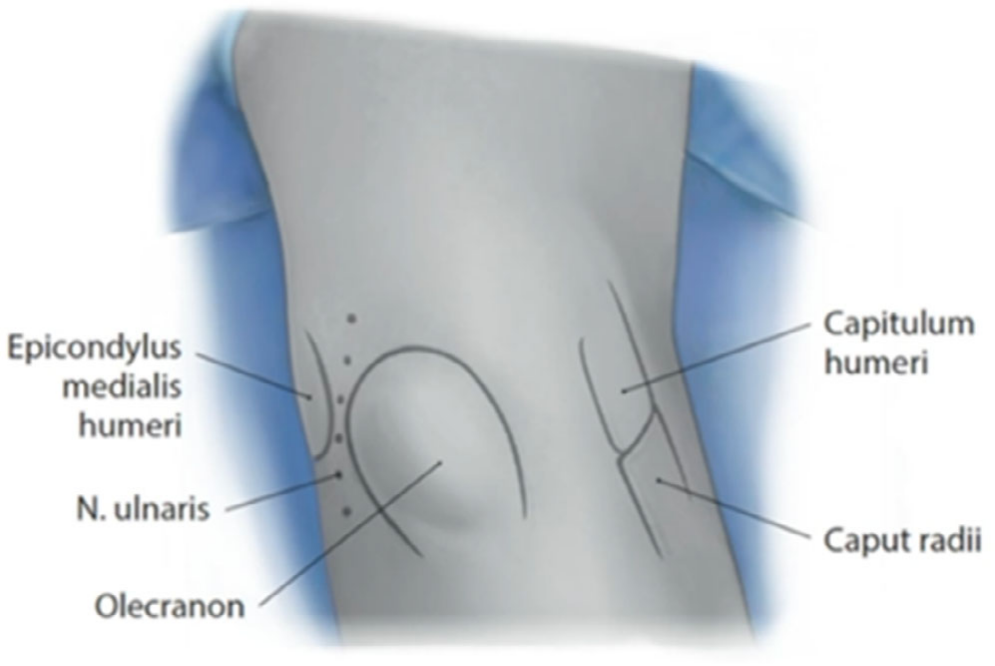
Anatomical landmarks. Key anatomical landmarks of the right elbow relevant for arthroscopy. The capitellum, radial head, and ulnar nerve are identified to facilitate orientation and safe portal placement.

Preoperative assessment includes a thorough clinical history and physical examination, as well as standard radiographs and magnetic resonance imaging to evaluate lesion size, stability, and associated intra‐articular pathology.^10^ The surgery can be performed with the patient under general or regional anesthesia. The patient is positioned in the lateral decubitus or prone position with the operative arm supported in an arm holder. The arthroscopy tower is placed at the head of the patient. After sterile preparation and draping, all relevant anatomical landmarks are marked (Figure 1). Venous blood is obtained from the patient at the beginning of the procedure and processed using a double‐syringe system to generate autologous conditioned plasma (ACP) according to the manufacturer's protocol (Arthrex, Munich, Germany). The key steps of the procedure are shown in Video 1.

### Portal Placement and Diagnostic Arthroscopy

**FIGURE 2:**
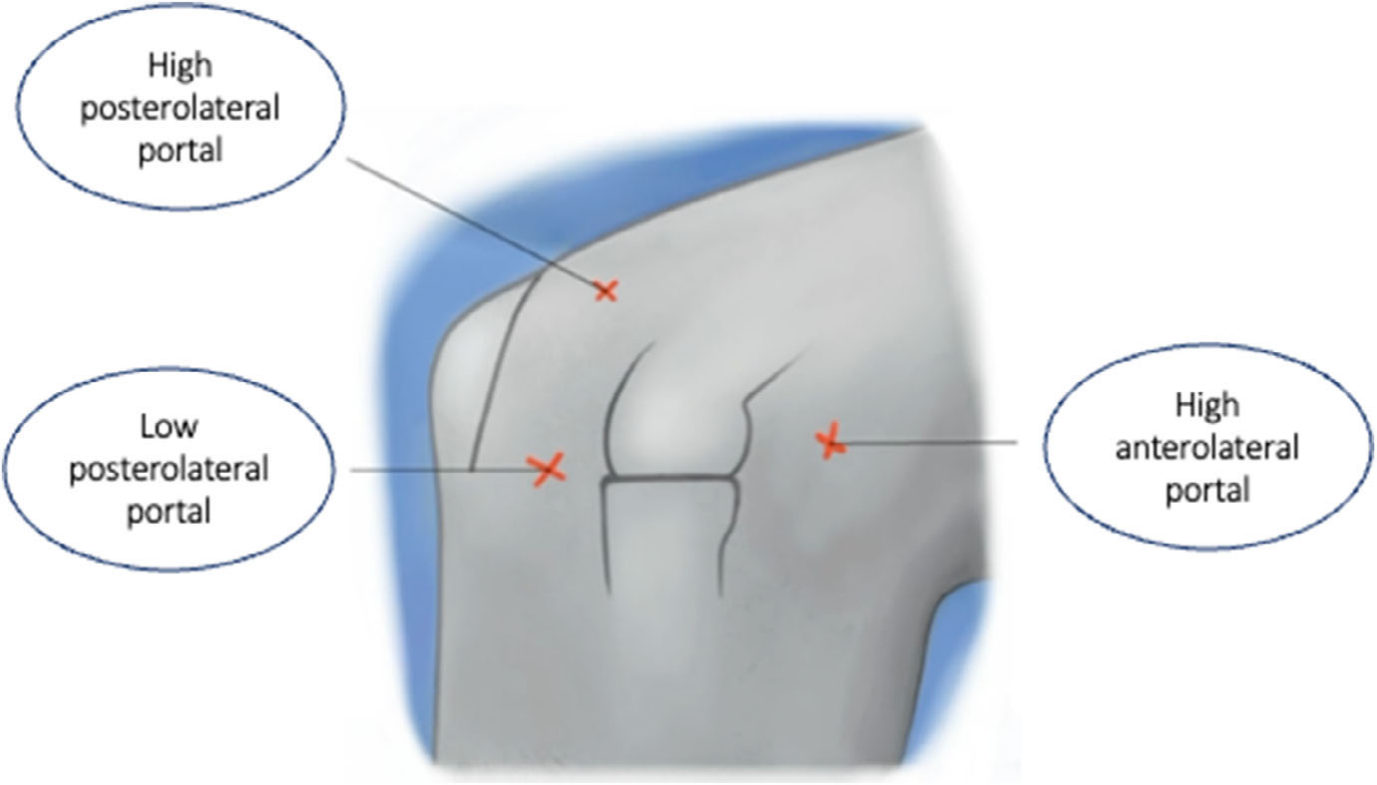
Portal placement. The right elbow is shown prior to arthroscopy with the skin marked to indicate standard portal placement. The anterolateral and posterolateral portals are identified relative to palpable anatomical landmarks, including the lateral epicondyle and radial head, to guide safe access to the radiocapitellar joint.

Arthroscopy is initiated through a high posterolateral viewing portal. A systematic inspection of the elbow joint is performed to identify concomitant pathology and to assess the osteochondral lesion. After rotating the arthroscope toward the radiocapitellar joint, hypertrophic tissue of the posterolateral plica, if present, can be visualized and carefully resected following establishment of a deep posterolateral working portal (Figure 2). This allows clear visualization of the capitellum humeri and radial head.

### Assessment of the Osteochondral Lesion

The osteochondral defect is carefully probed to assess fragment stability. In cases of fragment instability or complete detachment, refixation is not considered feasible based on fragment morphology and stability.

### Harvesting of Autologous Cartilage

**FIGURE 3:**
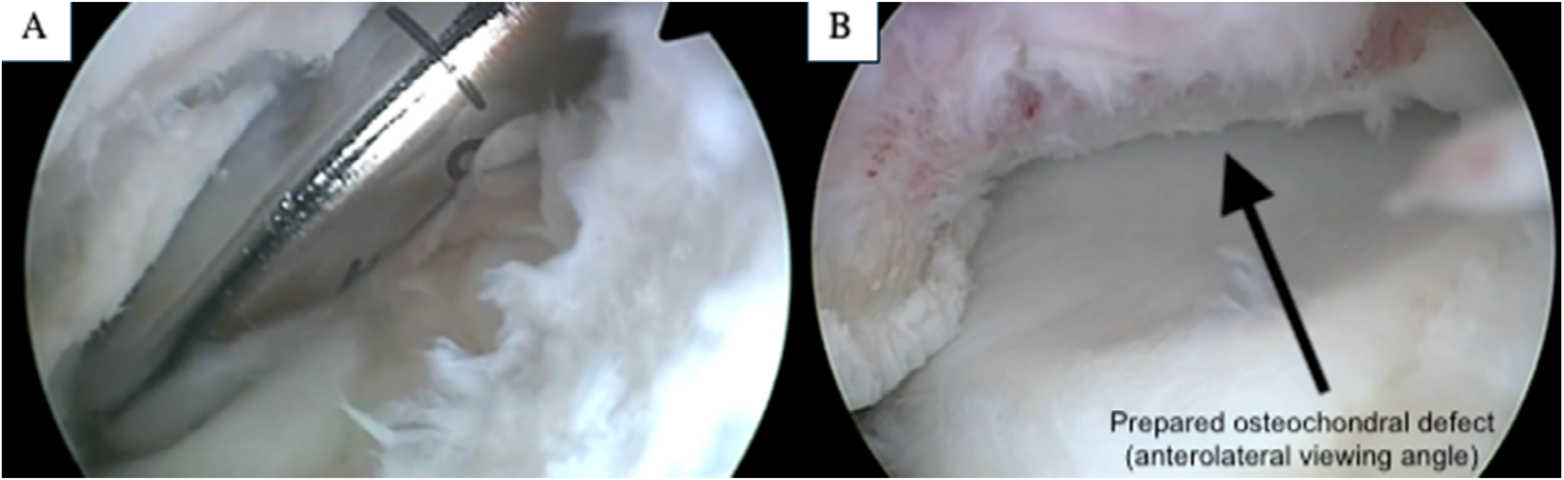
Harvesting of autologous cartilage and defect preparation. Arthroscopic views of the right elbow. After thorough visual inspection from the anterolateral portal and probing confirmed instability of the osteochondral fragment, refixation was considered unsuitable. Autologous cartilage was then harvested using a motorized shaver via the anterolateral portal, with careful detachment of the osteochondral fragment while preserving the subchondral bone (A). This allowed subsequent processing of the cartilage tissue outside the defect area. The prepared defect is illustrated from an anterolateral viewing angle (B).

Through the anterolateral and posterolateral portal, autologous cartilage tissue is harvested using a shaver while preserving the integrity of the subchondral bone. The osteochondral fragment is gradually mobilized under arthroscopic visualization. With alternating viewing from the high posterolateral portal and instrumentation through the low posterolateral portal, the fragment is completely released and removed arthroscopically using a grasping forceps. This allows subsequent preparation of the cartilage tissue outside the joint (Figure 3), using cartilage derived exclusively from unstable tissue removed during defect debridement, thereby avoiding additional donor site morbidity.

### Defect Preparation

The defect bed is further debrided through a low posterolateral or strictly lateral portal to create stable vertical cartilage margins, taking care not to violate the subchondral bone. Arthroscopic inflow is discontinued, and the joint fluid is evacuated via the low posterolateral portal. The defect is then meticulously dried using small swabs to optimize conditions for implantation (Figure 3).

### Preparation and Implantation of Minced Cartilage

**FIGURE 4:**
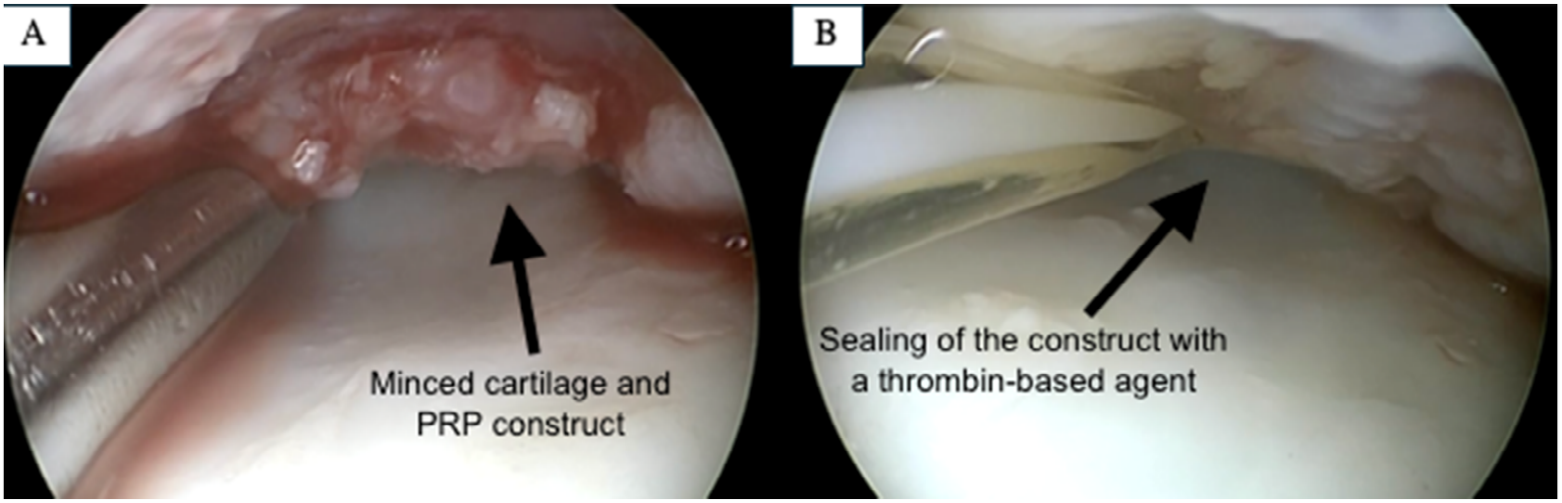
Minced cartilage implantation. Arthroscopic views of the right elbow. With visualization from the low posterolateral portal, the cartilage fragments were subsequently combined with autologous PRP and delivered into the defect through an application cannula. Using a blunt elevator, the graft was carefully contoured to achieve uniform defect coverage without exceeding the surrounding cartilage margins (A). An additional PRP layer was applied, followed by sealing of the construct with a thrombin‐based agent. Upon completion, the stability of the repair was confirmed through gentle passive elbow range‐of‐motion testing (B). (PRP, platelet‐rich plasma.)

Following cartilage harvesting and mechanical fragmentation, the minced cartilage particles are combined with the prepared ACP, creating a cohesive, paste‐like graft. The cartilage–platelet‐rich plasma mixture is delivered into the defect using an application cannula and gently molded with a blunt elevator to achieve homogeneous filling without overfilling the defect margins. After implantation of the cartilage–ACP mixture, thrombin is applied to induce fibrin formation through interaction with endogenous fibrinogen, thereby stabilizing the construct. Finally, the repair site is sealed with an additional fibrin layer generated by mixing ACP and thrombin in equal proportions, resulting in a stable cartilage repair construct after a short setting time of approximately 2 minutes. After completion of the implantation, gentle passive range‐of‐motion testing of the elbow is performed to confirm construct stability under dynamic conditions (Figure 4).

### Postoperative Care

Postoperatively, the elbow is immobilized at 90° in a cast for 24 hours. Thereafter, free range of motion exercises are allowed. Mechanical loading of the elbow should be avoided for 6 weeks postoperatively. After 3 months, progressive increase of load and sports‐specific training is initiated, with a targeted return to sport at 6 months postoperatively.

## DISCUSSION

This Technical Note describes a reproducible, step‐by‐step arthroscopic technique for autologous minced cartilage implantation in focal osteochondral lesions of the capitellum humeri. This joint‐preserving approach is gaining increasing attention, as it offers several potential advantages over established cartilage repair procedures, including a single‐stage application, avoidance of donor‐site morbidity, and biological restoration of the articular surface.

The elbow poses specific challenges for cartilage repair due to its thin cartilage layer, high joint congruency, and complex biomechanics. Arthroscopic access to the radiocapitellar joint can be technically demanding, particularly when addressing unstable osteochondral lesions.^11^, ^12^ A key benefit of this technique is the use of autologous cartilage harvested directly from the lesion itself or from detached osteochondral fragments, which are commonly encountered in advanced osteochondritis dissecans. This approach avoids the need for osteochondral graft harvest from distant donor sites and thereby eliminates donor‐site morbidity, a well‐recognized drawback of osteochondral autograft transfer procedures.^13^ Furthermore, the single‐stage nature of minced cartilage implantation simplifies surgical logistics and obviates the need for cell expansion or staged procedures^14^ (Table 1). Maintaining joint congruency is particularly critical in the elbow, where even small surface irregularities can result in pain, crepitus, or restricted motion. The described arthroscopic technique emphasizes stable defect margins, preservation of the subchondral plate, and avoidance of overfilling the lesion (Table 2).

**TABLE 1:** Advantages and Disadvantages of the Proposed Technique

**Advantages**	**Disadvantages**
• Single‐stage procedure	• Dependence on sufficient autologous cartilage tissue
• Avoidance of donor‐site morbidity	• Technical demand of cartilage handling and implantation
• Arthroscopic, minimally invasive approach	• Limited long‐term outcome data in the elbow

**TABLE 2:** Pearls and Pitfalls of the Proposed Technique

**Pearls**	**Pitfalls**
Thorough drying of the defect bed is critical for construct stability	Inadequate defect preparation
Avoid overfilling the defect margins	Excessive fluid contamination during implantation
Preserve subchondral bone integrity	Insufficient stabilization of cartilage fragments

From a biological perspective, minced cartilage implantation aims to promote hyaline‐like cartilage repair by transferring viable chondrocytes embedded within small cartilage fragments.^15^ These chondrocytes retain their native extracellular matrix environment, which is thought to support cell viability and matrix production.^16^ Unlike autologous chondrocyte implantation, the cells are not isolated or expanded ex vivo but remain within their physiological microenvironment, which may be advantageous for maintaining chondrogenic potential.^17^ Several experimental and clinical studies, especially for the knee, have shown that cartilage formed after minced cartilage implantation is comparable to repair tissue achieved with established cell‐based techniques and may be superior to marrow stimulation procedures.^18^, ^19^, ^20^, ^21^ Moreover, the size of the cartilage fragments plays an important role in the biological effectiveness of the technique. Small fragments of approximately 1 mm^3^ provide an optimal balance between surface area and cellular viability, facilitating chondrocyte migration and matrix synthesis. Standardized mechanical fragmentation using a 3‐mm shaver allows efficient preparation of these fragments without significantly compromising cell survival.^22^, ^23^

However, the procedure presented in this Technical Note is demanding and requires advanced arthroscopic skills. Thorough drying of the defect bed prior to implantation is essential to achieve adequate construct stability, as residual fluid may compromise adhesion of the cartilage–ACP composite. Preservation of the subchondral bone and stable defect margins is critical to maintain joint congruency, and overfilling of the defect should be avoided to prevent postoperative mechanical irritation (Table 2). In addition, adequate cartilage tissue must be available for fragmentation and implantation, limiting its applicability in cases of diffuse cartilage degeneration or bipolar lesions. Moreover, long‐term clinical and imaging outcomes following minced cartilage implantation in the elbow remain to be established (Table 1).

In conclusion, arthroscopic autologous minced cartilage implantation represents a feasible and biologically appealing option for the treatment of focal osteochondral lesions of the capitellum humeri. This Technical Note provides a step‐by‐step description of the surgical technique and may serve as a practical guide for surgeons seeking to expand their cartilage repair options in the elbow joint.

## DISCLOSURES

The author (L.P.M.) declares the following financial interests/personal relationships which may be considered as potential competing interests: L.P.M. reports consulting activities for Arthrex. The other authors (T.L., N.O., S.W., M.W., T.B.) declares that they have no known competing financial interests or personal relationships that could have appeared to influence the work reported in this paper.

## FUNDING

Open Access funding enabled and organized by Projekt DEAL.
